# Congenital Adrenal Hyperplasia and Human Leukocyte Antigen B: A Meta-Analysis

**DOI:** 10.7759/cureus.35900

**Published:** 2023-03-08

**Authors:** Dylan Thibaut, Madison R Walter, Courtney McGonegal, Ryan Daniel, Jerry Goodman

**Affiliations:** 1 Osteopathic Medicine, Lake Erie College of Osteopathic Medicine, Bradenton, USA; 2 Obstetrics and Gynecology, Lake Erie College of Osteopathic Medicine, Bradenton, USA

**Keywords:** non-classical congenital adrenal hyperplasia, congenital adrenal hyperplasia, mhc class i, hla-b, mhc, 21-hydroxylase deficiency, cah, haplotype, human leukocyte antigen, hla

## Abstract

The link between specific human leukocyte antigen (HLA)-B genes and congenital adrenal hyperplasia (CAH) has been a subject of interest. This study investigates the association between specific HLA-B haplotypes and CAH through a meta-analysis. Google Scholar was used as a database. Articles were included if the research was conducted between 1970 and 2022, was not a meta-analysis, and had odds ratios or enough data points to calculate an odds ratio. The National Institutes of Health (NIH) quality assessment tool of case-control studies was used to evaluate the risk of bias in individual studies, and MetaXL was used to generate data and create a forest plot for analysis. Twelve studies met the selection criteria and were included in the study (641 patients and 3,614 controls). Two HLA-B haplotypes showed increased odds of CAH compared to controls: B14 (OR=3.81; 95%CI=2.88, 5.05; I^2^=3%) and B35 (OR=1.88; 95%CI=1.22, 2.90; I^2^=25%). All other HLAs either showed no significant effect or had high heterogeneity. The results suggest that specific HLA-B haplotypes have increased odds of developing CAH, specifically B14 and B35. These findings may prove helpful in the pre- and post-natal diagnosis of CAH as well as the identification of carriers and prediction of patient prognosis.

## Introduction and background

Congenital adrenal hyperplasia (CAH) is an autosomal recessive disorder that affects approximately one in every 15,000 individuals [1]. CAH results from a deficiency in enzymes involved in cortisol and aldosterone biosynthesis. In roughly 90% of CAH cases, the most affected enzyme is 21-hydroxylase, an enzyme that is essential for synthesizing mineralocorticoids and glucocorticoids. In the absence of 21-hydroxylase, precursor-metabolite progesterone and 17-hydroxyprogesterone begin accumulating in high concentrations and are redirected toward the formation of adrenal androgens. This process initiates a feedback mechanism to stimulate the production of adrenocorticotropic hormone (ACTH) within the anterior pituitary gland through the hypothalamic-pituitary-adrenal axis, ultimately leading to bilateral adrenal fasciculata-reticularis zone hyperplasia, observed in all cases of CAH [2]. The physical manifestations of CAH depend on the severity of the enzymatic deficiency, resulting in a wide spectrum of clinical presentations.

One form of classical CAH is the salt-wasting (SW) form. This is the most severe form, resulting from a complete absence of 21-hydroxylase, and it typically presents with hyponatremia, hyperkalemia, and hypovolemia in both male and female infants [3]. Female infants present at birth with ambiguous genitalia because of hyperandrogenemia in utero. In contrast, male infants typically display more subtle manifestations, including slight skin hyperpigmentation and penile enlargement [3]. The second type of classical CAH is the simple-virilizing (SV) form. In this type, individuals have significantly low levels of 21-hydroxylase that allow for minute aldosterone production. As a result, the signs and symptoms exhibited correspond to the presence of ambiguous genitalia in female infants without life-threatening sodium deficiency. The clinical phenotypes overlap between the two classical CAH designations, as all patients lose salt and females with either form present with atypical genitalia. Non-classical CAH is a late-onset form, with clinical manifestations of amenorrhea, oligomenorrhea, hirsutism, and/or acne in females [3]. Previous studies have shown that not every individual with non-classical CAH is symptomatic; males are more likely to be asymptomatic and are usually identified after the diagnosis of a female family member [4]. Other enzymatic deficiencies, including the 11b-hydroxylase deficiency, also contribute to the development of CAH; however, these forms of CAH are observed less frequently.

Human leukocyte antigen (HLA)-B, a component of the class I major histocompatibility complex gene, is a major regulator of the immune system in preventing self-antigen recognition and autoimmunity. Recently, researchers have uncovered linkage disequilibrium associations between HLA genes and the 21-hydroxylase deficiency gene corresponding with the development of CAH, indicating that these genes may be inherited together. The 21-hydroxylase gene is in CYP21A2 and pseudogene CYP21P in chromosome 6p21.3 in the class III region of the major histocompatibility complex, placing it near both class I and class II major histocompatibility complex regions [5-8]. Various mutations, gene conversions, or deletions within either CYP21 or CYP21P in a homozygous recessive or compound heterozygous state cause CAH [8-11]. CYP21 gene expression occurs in the zona fasciculata and glomerulosa of the adrenal cortex, indicating that a deficiency involving these genes will impact these zones specifically [12]. Genetic mapping has not only linked these cytochromes to the C4A and C4B regions for C4 complement protein but also the HLA system [7,13]. It is also important to note that, of the possible enzyme deficiencies that result in CAH, only the 21-hydroxylase gene in CYP21A2 and pseudogene CYP21P are located in the class III region of the major histocompatibility complex; the 11b-hydroxylase gene is located in chromosome 8q, and the 17-hydroxylase gene is located in chromosome 10 [12].

This systematic review aims to determine if a linkage disequilibrium exists between specific HLA-B genes (HLA- B5, B7, B8, B12, B14, B15, B18, B27, B35, B40, and Bw47) and 21-hydroxylase deficiency. Establishing a link between HLA-B genes and CAH development may be a beneficial, specific genetic screening tool to detect individuals with minimal symptoms or maternal carriers and to determine whether further prenatal screening is necessary for the offspring of these individuals.

## Review

Methodology

The Preferred Reporting Items for Systematic Reviews and Meta-Analyses (PRISMA) guidelines were used to conduct the literature review [14]. To meet the eligibility requirements for this review, the study had to be written in English and conducted after 1970. Studies that included multiple measurements of different populations and meta-analyses or the ones that could not be accessed were excluded. Google Scholar was used as an information source. The exact search terms used in Google Scholar included “HLA-B” AND “CAH” and “HLA-B” AND “congenital adrenal hyperplasia.”

**Figure 1:**
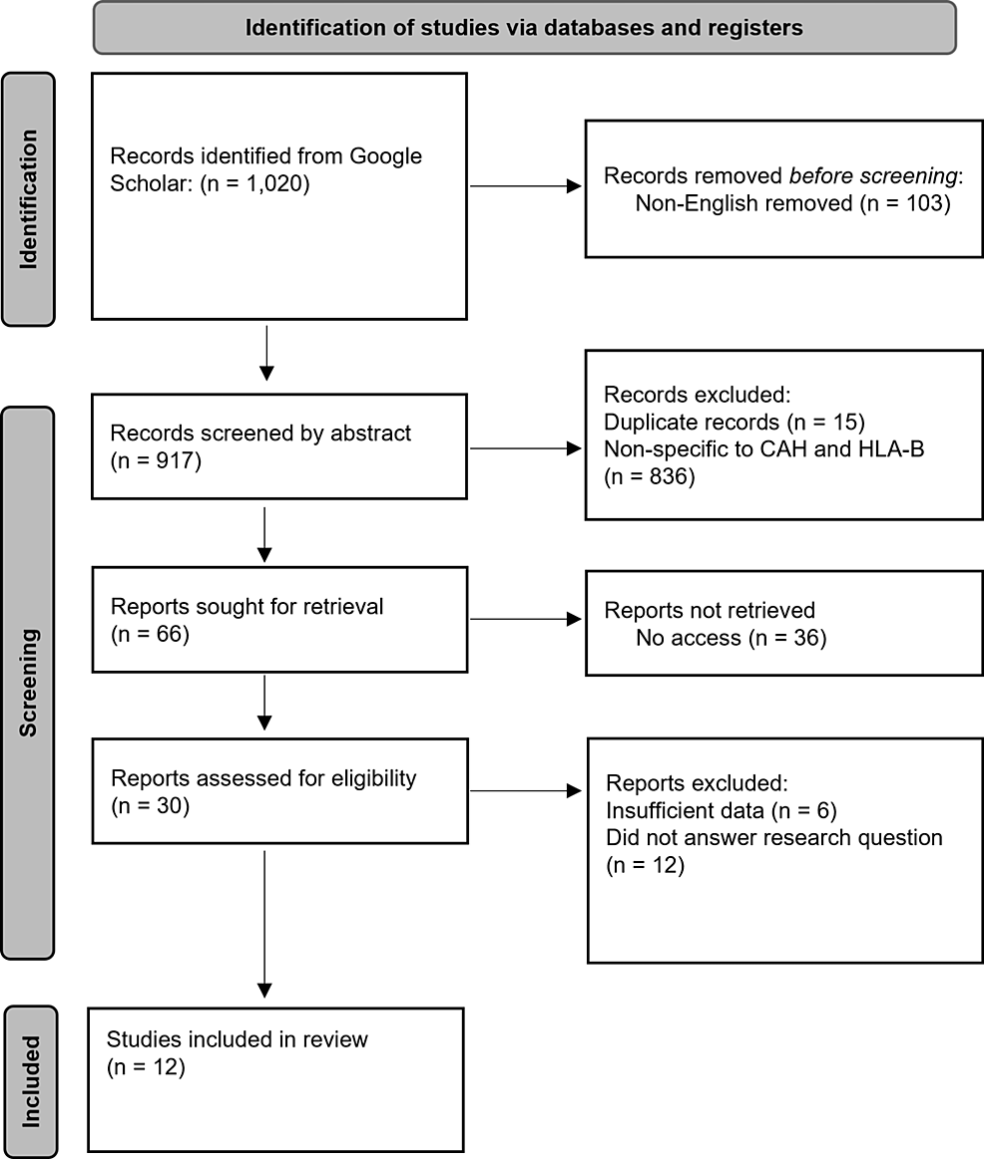
PRISMA flow diagram. HLA-B: human leukocyte antigen B; CAH: congenital adrenal hyperplasia; PRISMA: Preferred Reporting Items for Systematic Reviews and Meta-Analyses

Two independent researchers conducted the selection process and the data were compiled in a shared document. In the event of a disagreement regarding whether a study should move past the abstract screening, the researchers consulted the principal investigator. The results of this meta-analysis were calculated based on the data items collected from each selected study, including the number of cases of CAH with the specific HLA-B, total cases of CAH, control number of HLA-B, and the total number of controls. The odds ratios and confidence intervals were calculated with this data and used for analysis. The database search results and evaluation for inclusion criteria are shown in Figure 1 [14].

The effect size was assessed using odds ratios. Only studies that reported odds ratios or provided enough data to calculate the odds ratio were considered eligible and were included in the meta-analysis. Data were collected and compiled into tables for each HLA-B studied. RevMan 5.4 was used to generate a forest plot for each HLA-B to synthesize the data for the meta-analysis [15]. P-values below 0.05 provided enough evidence of a difference between the odds. I² was used to evaluate heterogeneity; a value below or equal to 25% was considered as evidence of low heterogeneity. Sensitivity analysis was completed using MetaXL [16]. The odds ratios obtained from the forest plot analysis and a 95% confidence interval were used to measure the outcome of this study. The National Institutes of Health (NIH) quality assessment tool of case-control studies was used to assess the risk of bias in individual studies [17]. A Doi plot and Luis Furuya-Kanamori (LFK index) were generated using MetaXL as a quantitative measure of bias [16].

Results

A total of 12 studies met the inclusion criteria for analysis [1,7,13,18-27]. Eleven HLA-Bs were selected for analysis: HLA-B5, B7, B8, B12, B14, B15, B18, B27, B35, B40, and Bw47. Table 1 summarizes the findings from the included studies as well as the bias assessment results.

**Table 1:** Summary of included studies and bias analysis. Results based on bias assessment tool [17] G: good; F: fair; P: poor; Y: yes; N: no; NA: not applicable; NR: not reported; CD: cannot determine

Study	Case total (N)	Control total (N)	Sample	Bias	1	2	3	4	5	6	7	8	9	10	11	12
Haghi Ashtiani et al., 2008 [1]	101	42	Iranian	G	Y	Y	N	Y	Y	Y	Y	NA	Y	Y	NR	NR
Klouda et al., 1980 [21]	48	230	Pakistinian & English	F	Y	N	N	NR	NR	NR	NR	CD	Y	Y	NR	Y
Couillin et al., 1980 [18]	52	106	French	G	Y	Y	N	N	Y	Y	N	NR	Y	Y	NR	Y
Pollack et al., 1981 [25]	36	4152	Ashkenazi & Non-Ashkenazi	G	Y	Y	N	N	CD	Y	NA	N	Y	N	NR	Y
Turowski et al., 1998 [27]	48	1152	Polish	G	Y	Y	NR	N	Y	NR	NA	N	Y	Y	NR	Y
Grubic et al., 2016 [20]	55	231	Croatian	F	Y	N	N	N	Y	Y	NA	N	NR	Y	NR	NR
Petersen et al., 1985 [24]	11	118	Eskimo	F	Y	Y	NR	Y	Y	Y	NA	NR	Y	Y	NR	NR
Morel et al., 1989 [7]	68	124	French Caucasian	G	Y	Y	N	NR	Y	Y	NA	NR	Y	Y	NR	N
Partanen et al., 1989 [23]	22	70	Finnish	F	Y	Y	NR	N	Y	Y	NA	N	Y	Y	NR	CD
Grosse-Wilde et al., 1979 [19]	27	1142	German	P	Y	N	N	NR	NR	Y	NA	N	Y	N	NR	Y
Knorr et al., 1985 [22]	163	4302	German	G	Y	Y	NR	Y	NR	Y	NR	N	Y	N	NR	Y
Speiser et al., 1985 [26]	314	2670	Ahskenazi, Hispanic, Yugoslav, Italian, Caucasians, Black	G	Y	Y	Y	Y	Y	Y	NA	NR	Y	Y	NR	Y

**Figure 2:**
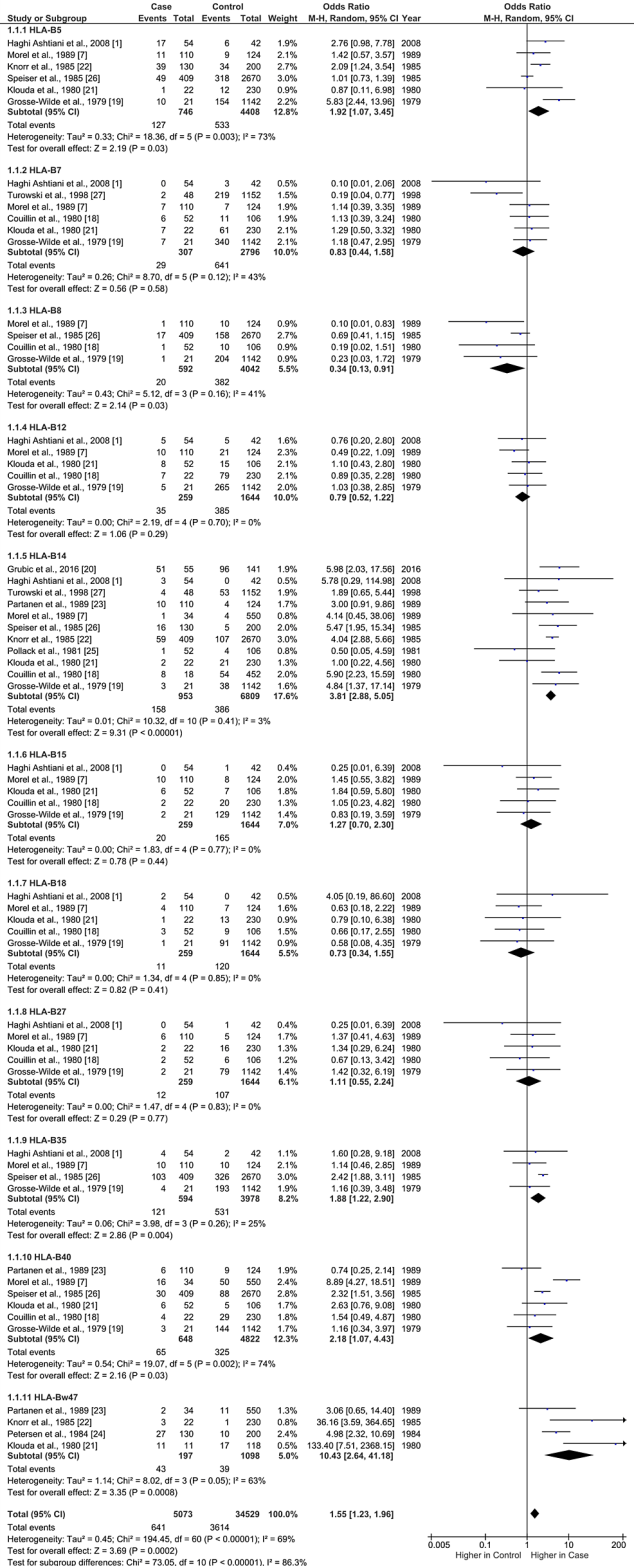
Forest plot results with associated statistics. HLA-B: human leukocyte antigen B Case: sample of CAH patients; control: sample of non-CAH patients

Meta-analysis results of each HLA-B as well as the overall analysis combining each group are listed in Table 2, and forest plot results with associated statistics are presented in Figure 2. Increased odds of the HLA and cases of CAH compared to controls with p<0.001 and I²≤25% include two HLA-B haplotypes: B14 (OR=3.81; 95%CI=2.88, 5.05) and B35 (OR=1.88; 95%CI=1.22, 2.90). All other HLA-B haplotypes did not show evidence of increased or decreased odds with low heterogeneity. B12, B15, B18, and B27 all had low heterogeneity but no findings of increased or decreased odds. All individual HLA analyses showed low heterogeneity (I²≤ 25%) except B5, B7, B8, B40, and Bw47. A combined analysis of all HLA-B haplotypes showed increased odds with high heterogeneity (OR=1.55, 95%CI=1.23, 1.96, I²=70%).

Heterogeneity amongst studies arises from low numbers of studies, small population size in certain studies, and the potential influence of different CAH types (non-classical, SW, etc.), which may show differences in frequency. Additionally, many studies under investigation were performed in the 1980s, with much lost to time outside of their abstracts, poor methods compared to updated methods, or inaccessibility online due to specifically needed journal subscriptions. This additionally may be a source of reporting bias. The certainty of the results is based on the collective evaluation of odds, confidence interval, bias, sensitivity, number of studies, p-value, and heterogeneity.

Regarding sensitivity analysis for the studies with the highest heterogeneity, Morel et al. was the greatest contributor in the B5 analysis, B40 was most influenced by heterogeneity due to support from Klouda et al., and Bw47 saw the greatest change due to support from Partanen et al. [7,21,23]. The B5 results contributed most to the heterogeneity when conducting the overall total combined analysis. These results are summarized in Table 2.

**Table 2:** Meta-analysis results. *I²≤25%, †p<0.001, ‡p<0.05 LFK: Luis Furuya-Kanamori; HLA: human leukocyte antigen Case: sample of CAH patients; control: sample of non-CAH patients

HLA	Case with HLA (N)	Case Total (N)	Control with HLA (N)	Control total (N)	OR (95%CI)	Odds	I²	LFK index	Most contributing cause of increased heterogeneity
B5‡	127	746	533	4408	1.92 (1.07, 3.45)	↑	73%	1.94	Morel et al., 1989 [7]
B7	29	307	641	2796	0.83 (0.44, 1.58)	↔	43%	-3.49	Couillin et al., 1980 [18]
B8‡	20	592	382	4042	0.34 (0.13, 0.91)	↓	41%	-5.83	Grosse-Wilde et al., 1979 [19]
B12*	35	259	385	1644	0.79 (0.52, 1.22)	↔	0%	-0.42	
B14*†	158	953	386	6809	3.81 (2.88, 5.05)	↑	3%	-1.26	Morel et al., 1989 [7]
B15*	20	259	165	1644	1.27 (0.70, 2.30)	↔	0%	-3.30	
B18*	11	259	120	1644	0.73 (0.34, 1.55)	↔	0%	2.30	
B27*	12	259	107	1644	1.11 (0.55, 2.24)	↔	0%	-3.22	
B35*‡	121	594	531	3978	1.88 (1.22, 2.90)	↑	25%	-5.78	Haghi Ashtiani et al., 2008 [1]
B40‡	65	648	325	4822	2.18 (1.07, 4.43)	↑	74%	-1.12	Klouda et al., 1980 [21]
Bw47†	43	197	39	1098	10.43 (2.64, 41.18)	↑	63%	3.88	Partanen et al., 1989 [23]
Total†	641	5073	3614	34529	1.55 (1.23, 1.96)	↑	69%	-1.43	

Major asymmetry of the found LFK index and Doi plot data in Table 2 is LFK index greater than +2 or less than -2. Asymmetry may hint at possible bias influencing the results of the analysis. Studies of the HLA genes B7, B8, B15, B18, B27, B35, and Bw47 showed major asymmetry. Analysis of HLA genes B5, B12, B14, and B40 and the combined analysis of all HLA-B did not show major asymmetry.

The NIH quality assessment tool of case-control studies was used to assess each study’s internal validity (Table 1). Seven of the 12 studies included in the meta-analysis had good internal validity, and four had fair internal validity. One study, Grosse-Wilde et al., had poor internal validity due to poorly defined demographics, inconsistent methods between participants, and lack of sample size justification and concurrent controls [19]. This study also did not report their methods of patient recruitment, inclusion criteria, exclusion criteria, or whether the assessors were blinded to the case or the control status of the participants.

Discussion

Previous studies have proven a close genetic linkage between HLA and CAH [5-13]. Our meta-analysis focused on evaluating which of the HLA-B genes had the strongest association with the occurrence of CAH. The results showed an increased odds ratio for HLA-B and the presence of CAH in two of the 11 HLA-B genes selected, indicating a higher association between the HLA-B alleles and CAH. These included B14 (OR=3.81; 95%CI=2.88, 5.05) and B35 (OR=1.88; 95%CI=1.22, 2.90). Both were statistically significant (p<0.05) with low heterogeneity. This means that there was low variability in the data. B12, B15, B18, and B27 had low heterogeneity but were not found to have any effect on CAH. All the other HLA-B alleles studied had moderate to high heterogeneity.

There are a few limitations to consider in the HLA typing samples included in this analysis. Most of the data retrieved were obtained from research studies conducted in the 1980s. This created limitations in the review process by preventing full access to all articles due to their age. Additionally, with over 2000 alleles at the HLA-B locus, it was not practical or time-efficient to conduct a meta-analysis to cover them all [28]. We set a cap of 11 HLA-B alleles to study and went in order of HLA until the cap was met. Our data was also limited because we only used Google Scholar rather than multiple databases. This was done to further narrow down the number of HLA-B alleles to be included in the study.

Demonstrating a genetic linkage between HLA-B genes and the 21-hydroxylase deficiency has provided an additional diagnostic tool in both pre- and postnatal cases [29]. Based on our meta-analysis, HLA typing of amniotic cells that reveals HLA-B14 and HLA-B35 may prompt further evaluation for CAH in prenatal cases to prevent any delays in treatment. HLA-B typing for these specific genes should also be considered for newborns of mothers who did not undergo amniotic screening. Additional hormonal studies will be useful to confirm a diagnosis of CAH and rule out false positives. Screening prospective parents can be clinically relevant in terms of genetic counseling.

With heterozygote carriers and those who present with nonclassical CAH expressing few to no symptoms, HLA-B screening may be useful in identifying such carriers. The identification of a carrier may prompt clinicians to obtain maternal serum for cell-free fetal DNA sequencing, a technique that can correctly deduce fetal CAH as early as five weeks and six days of gestation [30-31]. Advantages of early diagnosis and treatment include, but are not limited to, avoiding severe SW that can progress to adrenal crisis, genital ambiguity, and precocious puberty. Aside from the physical manifestations of CAH, prompt and effective treatment can help avoid gender confusion and psychosexual disturbances in both affected boys and girls [32-34]. Prenatal treatment involves the immediate use of dexamethasone in early pregnancy (beginning by nine weeks gestation), which has shown success but must be given with caution [35,36]. Postnatal treatment involves using glucocorticoids and mineralocorticoids depending on symptom severity as the phenotype of CAH presents on a continuum. Further research investigating if the presence of these HLA-B genes can correctly predict what subtype of CAH a patient will present with may prove to be valuable in the management of future cases.

## Conclusions

The goal of this study was to assess the strength of evidence for an association between several HLA-B alleles and CAH. The findings report two main things. HLA-B14 (OR=3.81; 95%CI=2.88, 5.05) and B35 (OR=1.88; 95%CI=1.22, 2.90) have increased odds of CAH. HLA-B12, B15, B18, and B27 had low heterogeneity but were not found to have any effect on CAH. Overall, this result indicates that several HLA alleles are linked to CAH while others are not. This result allows future research to focus on HLA-B14 and HLA-B35 to find the genetic reasons behind their linkage with CAH, possibly generating diagnostic or prognostic implications.
